# Granulocyte Colony Stimulating Factor Induces Lipopolysaccharide (LPS) Sensitization via Upregulation of LPS Binding Protein in Rat

**DOI:** 10.1371/journal.pone.0056654

**Published:** 2013-02-20

**Authors:** Haoshu Fang, Anding Liu, Jian Sun, Alexandra Kitz, Olaf Dirsch, Uta Dahmen

**Affiliations:** 1 Experimental Transplantation Surgery, University Hospital of Jena, Jena, Germany; 2 Experimental Medicine Center, Tongji Hospital, Huazhong University of Science and Technology, Wuhan, China; 3 Department of Hepatobiliopancreatic Surgery, Sun Yat-sen Memorial Hospital, Sun Yat-sen University, Guangzhou, China; 4 Institution of Neuroimmunology, Georg-August-University Gottingen, Gottingen, Germany; 5 Institute for Pathology, University Hospital of Jena, Jena, Germany; INSERM, France

## Abstract

Liver is the main organ for lipopolysaccharide (LPS) clearance. Sensitization to LPS is associated with the upregulation of LPS-binding protein (LBP) in animal models. Therefore, we hypothesized that LBP could induce LPS sensitization through enhancing hepatic uptake of LPS. In this study, we examined the role of LBP in pathogenesis of LPS induced systemic inflammatory response syndrome (SIRS). LBP expression was upregulated after granulocyte colony stimulating (G-CSF) pretreatment. The effect of LBP was further confirmed by blockade of LBP using LBP blocking peptide – LBPK95A. After G-CSF pretreatment, upregulation of LBP was observed in bone marrow cells and liver. The G-CSF induced LBP upregulation caused LPS hypersensitization in rats as indicated by higher mortality and severer liver damage. Of note, LBP blockade increased the survival rate and attenuated the liver injury. The LBP induced LPS hypersensitization was associated with increased hepatic uptake of LPS and augmented hepatic expression of LPS receptors, such as toll-like receptor (TLR)-4. Furthermore, LBP mediated early neutrophil infiltration, which led to increased monocyte recruitment in liver after LPS administration. In conclusion, G-CSF induced LBP expression could serve as a new model for investigation of LPS sensitization. We demonstrated the crucial role of LBP upregulation in pathogenesis of LPS induced SIRS.

## Introduction

Sepsis is a major and growing health problem, which is frequently associated with increased blood levels of lipopolysaccharide (LPS) [1]. LPS is a constituent of the outer cell wall of gram-negative bacteria. LPS leads to a dose dependent inflammatory response of the organism, ultimately resulting in the systemic inflammatory response syndrome (SIRS), LPS shock and death of the organism. Therefore, LPS is considered to be the most important bacterial factor in the pathogenesis of SIRS and sepsis [2].

Sensitization to LPS was observed in different animal models. Sensitization to LPS is characterized by an enhanced, even lethal inflammatory response subsequent to a moderate challenge with LPS. It was reported that hemorrhagic shock led to enhanced sensitivity to LPS [3]. Minter et al reported that common bile duct ligation (BDL) also induced LPS hypersensitization [4]. This phenomenon was observed as well after pretreatment with galactosamine [5], propionibacterium parvum [6], bacillus calmette-guerin [7] and CpG DNA [8].

LPS-binding protein (LBP) is elevated after LPS treatment. LBP is a 55 kDa acute phase protein mainly produced by liver [9]. LBP is constitutively expressed at low levels and is upregulated during acute phase responses [10]. In vivo, LPS is firstly recognized by LBP, and then transferred to its receptor toll-like receptor (TLR) 4 and triggers an inflammatory response [11]. Because of the LPS binding activity, LBP is considered to play an important role in mediating the inflammatory response. Elevation of peripheral LBP levels is widely observed in clinical settings, and moderately correlated with the severity of disease [12], [13]. The inflammatory response to LPS can be reduced by interfering with the interaction of LPS and LBP. Experimental strategies include using LBP-inhibitory peptides [14], LBP-antibody [15], or LBP knockout mice (LBP-KO) [16], [17].

We recently observed that granulocyte colony-stimulating factor (G-SCF) pretreatment caused upregulation of LBP [18]. G-CSF is an interesting substance since it is known to upregulate innate immunity [19], e.g. by promoting the proliferation of neutrophils and release into the peripheral blood. G-CSF was already used successfully to treat experimentally induced sepsis using the cecal ligation and puncture model in rats [20]. However, clinical trials where G-CSF was used therapeutically to treat ongoing severe sepsis or given as prophylactic treatment did not result in a clear benefit [21].

Of note, in our previous study, LBP was upregulated after G-CSF pretreatment in vivo [18]. These data indicated that G-CSF might increase the expression of LBP, thereby playing an important role in modulating the inflammatory response. We hypothesized that G-CSF may cause sensitization to LPS via upregulation of LBP.

The mechanism underlying LBP-mediated LPS-sensitization is not well elucidated. Since LBP is an acute phase protein synthesized by liver, we hypothesized that the sensitization to LPS may result from an enhanced LPS-binding to the liver via upregulating hepatic LBP expression.

## Materials and Methods

### Experimental Design

The distribution of each experimental group was described in Table 1. To confirm whether G-CSF could upregulate LBP expression, rats were pretreated with G-CSF (100 µg/kg/day, subcutaneous injection, ratiopharm, Breda, Netherland) for 5 days (G-CSF group). To investigate whether G-CSF could induce sensitization to LPS, rats were challenged with a sub-lethal LPS injection (2 mg/kg, intravenous injection, *E. coli* serotype O55:B05 type, Sigma-Aldrich, St. Louis, USA) only (LPS group) or 1 day after G-CSF treatment (G-CSF+LPS group). To further confirm the function of G-CSF induced LBP expression, LBP inhibitory peptide LBPK95A (5 mg/kg, RVQGRWKVRASFFK, synthesized in-house using an Fmoc standard procedure on an ABI 433A-peptide-Synthesizer), was injected intraperitoneally 2 h before LPS administration (G-CSF+LPS+LBPK95A group) [4]. Serum and hepatic LBP levels, mortality, hepatic injury, hepatic uptake of LPS, inflammatory response, neutrophil infiltration and monocyte recruitment was investigated.

**Table 1:** Group distribution.

Group (n = 6/group)	Treatment			Observation time
	G-CSF	LPS	LBPK95A	
LPS	−	+	−	1 h, 6 h, 24 h
G-CSF+LPS	+	+	−	1 h, 6 h*
G-CSF+LPS+LBPK95A	+	+	+	1 h, 6 h, 24 h

* In G-CSF+LPS group, no rats could survive longer than 6 h.

To investigate whether G-CSF could enhance LPS binding to the liver, the kinetics of hepatic LPS uptake was observed 10 min, 30 min and 60 min after LPS administration (2 mg/kg, n = 3 per group) with or without G-CSF pretreatment and/or LBP blockade.

### Animal Model

Male inbred Lewis rats (250–350 g, Charles River, Sulzfeld, Germany) were used in this study. All animals were housed under standard animal care conditions and had free access to water and rat chow ad libitum. All procedures were carried out according to the German Animal Welfare Legislation, and were performed under inhalation anesthesia with 1.5%–3.0% isoflurane (Sigma Delta, London, UK).

### Isolation of Rat Bone Marrow Cells, Peripheral Macrophage and Peripheral Blood Mononuclear Cells (PBMC)

Bone marrow cells were harvested from femurs of rats by two times washings with 10 ml PBS (3 U/ml heparin). After lysis of red blood cells and 2 time washing (300 g×3 min), the cells were re-suspended in PBS and smears were prepared. The macrophage and PBMC was isolated as described previously [22].

### Liver Enzyme

Liver injury was investigated by measuring the serum level of aspartate transaminase (AST) using an Automated Chemical Analyzer (Bayer Advia 1650; Leverkusen, Germany).

### Histological Staining

Liver tissue was fixed in 4.5% buffered formalin for at least 24 h. Paraffin embedding was performed, and sections (4 µm) were cut and stained with Hematoxylin-Eosin (HE). Slides were digitalized using a virtual slide scanner (Hamamatsu Electronic Press Co., Ltd, Lwata, Japan). Histological evaluation was performed according to a standardized semi-quantitative scoring system (Table 2, Figure S1).

**Table 2:** Parameters of semi-quantitative scoring system for histological evaluation.

	1	2	3	4
Alteration in sinusoids	No dilatation and cell accumulation	Dilatation of sinusoids or cell accumulation	Dilatation and cellaccumulation	Severe dilation and cell accumulation
Vacuolization of hepatocytes	Not present	1%–10% of all the hepatocytes	10%–30% of all the hepatocytes	>30% of all the hepatocytes
Erythrocyte congestion	No erythrocyte congestion	Minor erythrocyte congestion	Moderate erythrocyte congestion	Severe erythrocyte congestion
Hepatocellular necrosis	No necrotic hepatocytein 5 HPF (40X)	1–10 in 5 HPF	10–30 in 5 HPF	>30 in 5 HPF

### LBP IHC Staining

Antigen retrieval and Nonspecific protein binding were performed as described above. Sections were incubated with monoclonal rat anti-LBP antibody (1∶100, cell science, Canton, USA) for 1 h. Normal rat IgG (Sigma-Aldrich, St.Louis, USA) was used as a negative control. Detection was performed using bright vision rabbit-anti-mouse-AP (ImmunoLogic, Duiven, Netherlands) and employing fast-red (Abcam, Cambridge, UK) signal detecting system. Slides were digitalized as described above.

### LPS IHC Staining

After deparaffinization and rehydration, antigen retrieval was performed using citrate buffer (10 mM Citric Acid, pH 6.0) for 20 min at 100°C. Nonspecific protein binding was blocked using 100 µl serum free blocking buffer (Dako, Glostrup, Denmark). Sections were incubated with polyclonal mouse anti-LPS antibody (1∶100, Abcam, Cambridge, UK) for 15 min. Signals were amplified using CSA II biotin-free tyramide signal amplification system (Dako, Glostrup, Denmark). Sections were counterstained with Hematoxylin for 5 min and digitalized as described above.

### Naphthol-AS-D-chloroacetate Esterase (ASDCL) Staining

Neutrophil infiltration into liver tissue was evaluated by ASDCL staining as reported previously [23]. After staining, slides were visualized using the virtual slide scanner, and 5 high-power fields (HPF) pictures were randomly taken with magnification of 200×. ASDCL staining positive neutrophils were counted manually. The result was expressed as the number ASDCL positive neutrophils per HPF.

### Electrophoresis and Western Blot (WB)

Fifteen µg of the total liver lysate protein or 3 µl serum were loaded per well and separated on 12% gels by sodium dodecyl sulfate-polyacrylamide gel electrophoresis followed by western blotting and staining with rat anti-LBP antibody (1∶100, Santa Cruz Biotechnology, Santa Cruz, USA), anti-macrophage cationic peptide (MCP) 1 antibody (1∶2000, abcam, Cambridge, UK), anti-macrophage inflammatory protein 1 alpha (MIP-1α) antibody (1∶400, abcam, Cambridge, UK) and glyceraldehyde-3-phosphate dehydrogenase (GAPDH) antibody (1∶20000, Sigma-Aldrich, St. Louis, USA). Signals were detected with Lumilight western blot substrate (Roche, Basel, Switzerland) and exposed to X-ray film (GE Healthcare, Buckinghamshire, UK). The signal intensity was quantified with ImageJ 1.43 G (NIH, Bethesda, USA).

### Quantitative Polymerase Chain Reaction (PCR)

Total RNA was extracted from liver tissue using the RNeasy kit (Qiagen, Hilden, Germany). 2–5 µg of total RNA from each sample was used for cDNA synthesis using the First-Strand cDNA synthesis kit (Invitrogen, Carlsbad, USA). An equal amount (1 ng) of cDNA was used for each quantitative PCR reaction. PCR reaction mixture was prepared using Brilliant probe-based QPCR Master Mix kit (Agilent, Santa Clara, USA), probes (Universal Probe Library) and primers (Table S1). The reaction mixtures were incubated at 95°C, followed by 50 cycles of 95°C for 30 s, 50°C for 30 s and 72°C for 30 s in M×3000P QPCR System (Stratagene, La Jolla, USA). Standard curve was generated using a serial dilution of a normal sample. Gene expression was normalized using hypoxanthine-guanine phosphoribosyltransferase (HPRT) to compensate for errors when diluting the cDNA stock solution. The fold change was calculated with the gene expression using the liver tissue samples 1 h after LPS injection as reference sample.

### Enzyme-linked Immunosorbent Assay (ELISA)

Serum IL-6 and TNF-α level were determined with commercial ELISA kits (R&D Systems, Minneapolis, US). All procedures were performed according to the instructions of the manufacturer. Measurements of the ELISA were performed in 96-well polysterene plates using an ELISA plate reader (Bio-Tek Instruments Inc., Winooski, VT, US) at 450 nm.

### Statistical Analysis

All values were expressed as mean ± SD. All statistical calculations were performed by using Sigma Stat (ver. 3.5.54; Systat Software GmbH, Erkarth, Germany). Groups of animals were compared employing Student’s t-test in case of normal distribution of the data. If data were not normally distributed, the Mann-Whitney rank sum test was employed to compare sets of data in different experimental groups. A p-value below 0.05 was considered statistically significant.

## Results

### G-CSF Pretreatment Induces LBP Expression

**Figure 1:**
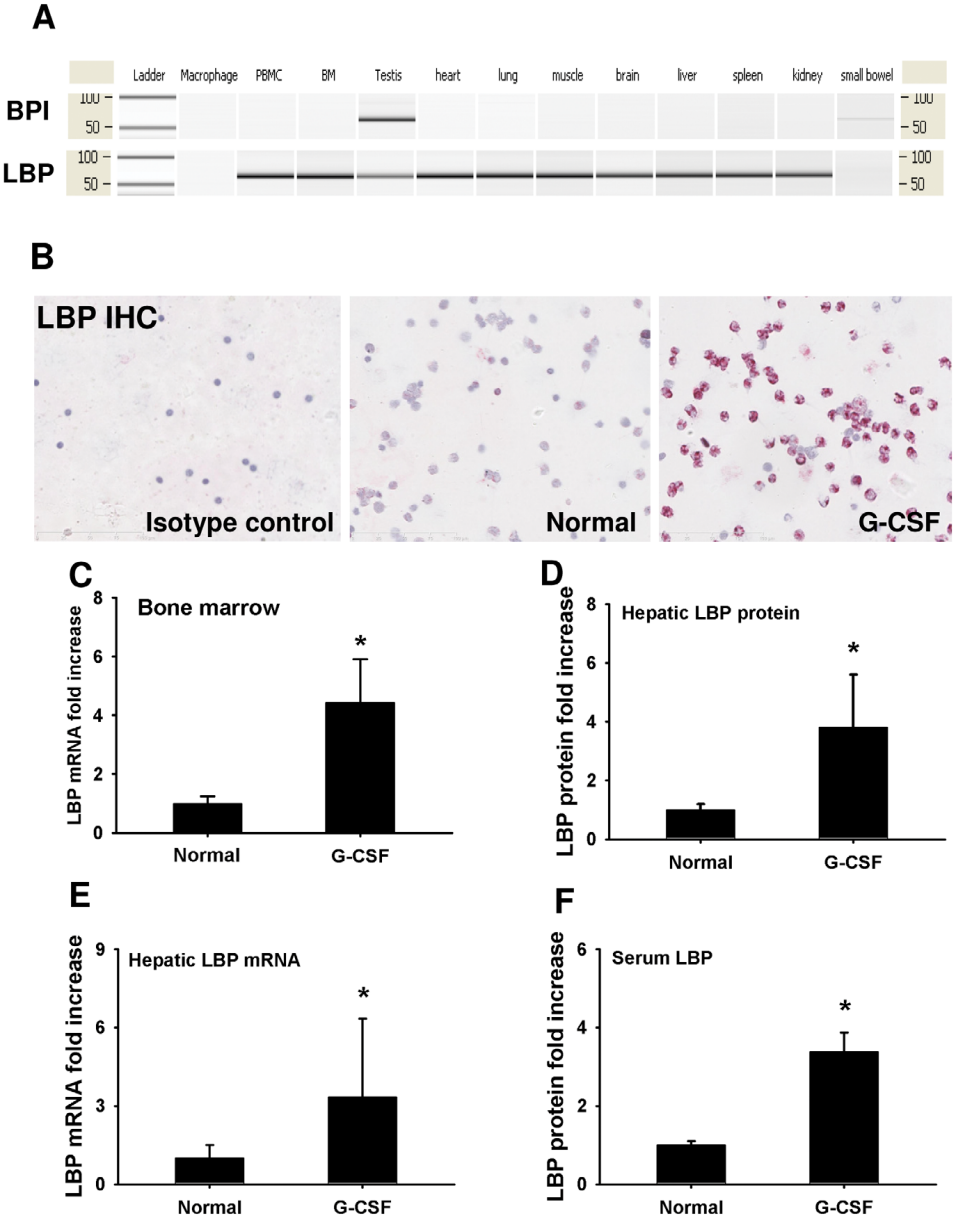
G-CSF pretreatment induced the expression of LBP in granulocytes and liver. (**A**) Expression of LBP and BPI was detected by quantitative PCR in various cells and organs of rats. The BPI mRNA was only detectable in testis. In contrast, the gene expression of LBP was detected universally in most of the organs and cells except macrophage. (**B**) Results from LBP IHC staining shown that expression of LBP protein was upregulated in bone marrow cells after G-CSF pretreatment. Original magnification ×200. (**C**) LBP mRNA levels were increased significantly in bone marrow cells after G-CSF pretreatment (p<0.001). (**D–F**) Hepatic LBP mRNA, protein and serum LBP levels were measured using quantitative PCR and western blot. Both LBP mRNA and protein levels were upregulated significantly after G-CSF pretreatment. *p<0.05 vs. normal control. Data are shown as mean ± SD, n = 6 per group.

To investigate the effect of G-CSF pretreatment on LBP expression, we tested LBP expression after G-CSF pretreatment. To exclude the potential cross-reaction between LBP and BPI, we first analyzed the expression of LBP and BPI in different organs by quantitative PCR (Figure 1A). The BPI mRNA was highly expressed in testis, but was undetectable in other organs as described before [24]. In contrast, the LBP is expressed universally in most of the cells and organs except macrophages (Figure 1A). LBP IHC staining showed an increase of LBP protein expression in BMC after G-CSF pretreatment (Figure 1B). Consistently, a similar result was observed by quantitative PCR (Figure 1C, p<0.001). Furthermore, both hepatic and serum LBP-protein levels were increased by about 3-fold as shown in Figure 1D–F. Due to the unique LPS binding ability of LBP, we hypothesized that LBP might play an important role in mediating the LPS induced inflammation and neutrophil infiltration.

### G-CSF Induced LBP Expression Sensitizes to a Subsequent LPS Challenge

**Figure 2:**
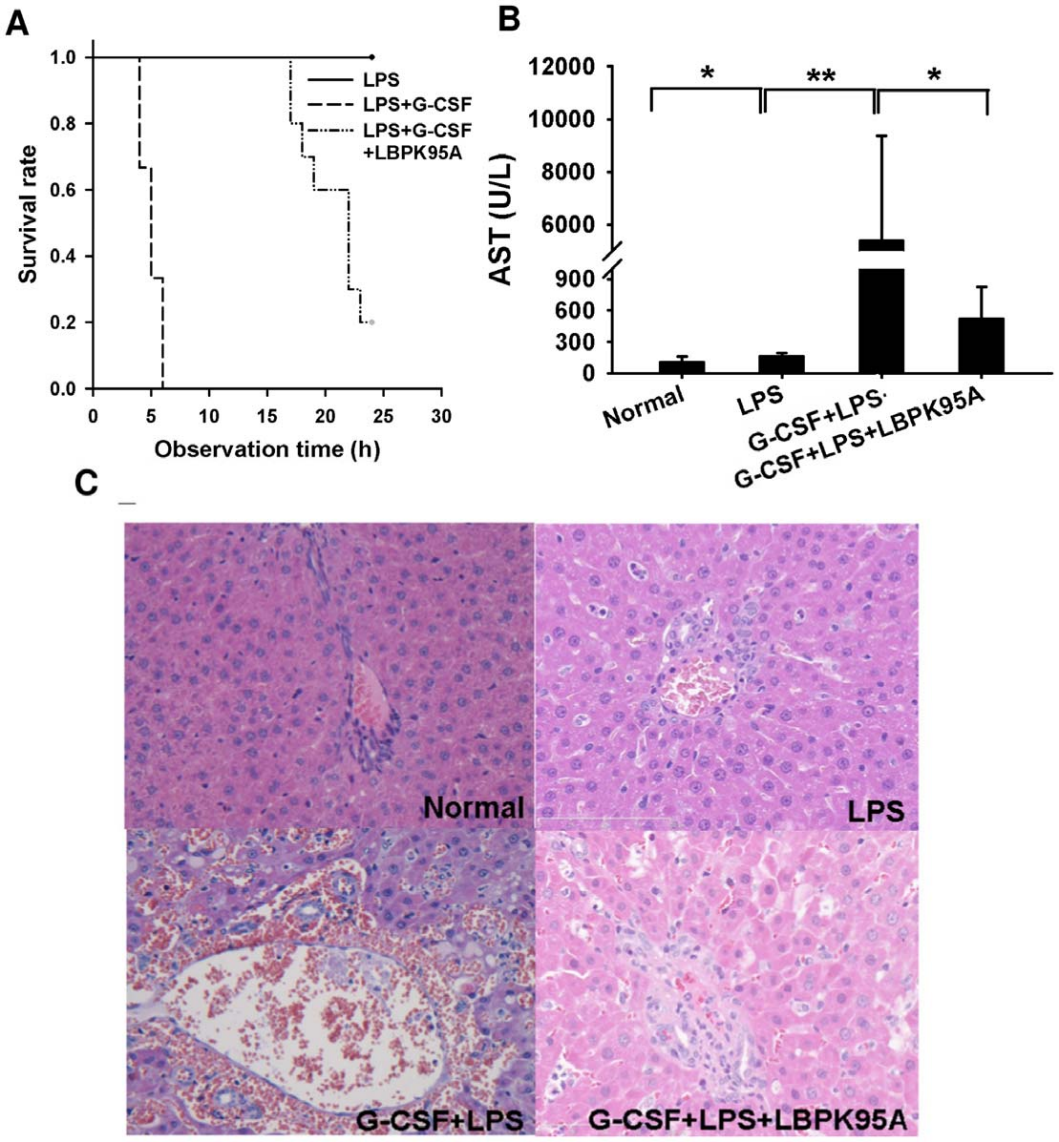
G-CSF induced LBP expression sensitized to LPS. (A) Pretreatment of G-CSF caused 100% mortality within 4–6 h after LPS injection in G-CSF+LPS group. Survival rate was significantly increased in rats after blockade of LBP using LBP blocking peptide – LBPK95A (p<0.001). (B) Serum AST levels were analyzed to assess hepatocellular injury 6 h after LPS injection. Blocking the elevated LBP prevented hepatic damage as indicated by lower serum AST levels. (C) Rats with G-CSF pretreatment and LPS injection showed severe histological damage such as sinusoid dilation, and erythrocyte congestion. Rats in G-CSF+LPS+LBPK95A group showed less damage and minimal histopathologic changes (HE staining). Representative images from 6 rats per group were selected. Original magnification ×200. *p<0.05, **p<0.01 Data are shown as mean ± SD, kinetic experiment: n = 6 per group; survival experiment: n = 10 per group.

We demonstrated that G-CSF pretreatment induced LBP expression. Next, we assessed the LPS-response in animals, which presented with elevated LBP-levels after G-CSF pretreatment. The G-CSF induced LBP expression caused the death to a subsequent LPS challenge in 10/10 rats within 24 h. All control rats developed fever and showed signs of sickness behaviours, but survived after LPS administration (2 mg/kg). In contrast, in the G-CSF pretreatment group, all rats died within 6 h after LPS challenge (Figure 2A). Therefore, we suspected that the mortality might be associated with the G-CSF induced LBP expression. To test this, we blocked LBP using LBP blocking peptide-LBPK95A in vivo, and found that blockade of LBP significantly increased the survival rate and prolonged the survival time (p<0.001). Moreover, the results of liver damage were paralleled with the survival data. As shown in Figure 2B, severe liver injury indicated by a highest serum AST levels was observed in G-CSF+LPS group at 6 h after LPS administration. In contrast, serum AST levels were significantly reduced in rats treated with LBP inhibitory peptide LBPK95A (Figure 2B, p<0.005). The serum AST elevation, indicative of liver damage, was confirmed by the histological evaluation. Severe erythrocyte congestion, sinusoidal dilation and lymphocytes infiltration were present in liver tissues obtained from G-CSF+LPS group at 6 h after LPS injection (Figure 2C; Table 3). Pretreatment with LBP inhibitory peptide resulted in less liver damage, indicating that LBP blockade protected the liver against LPS induced injury. These data suggested that, G-CSF induced LBP expression in rats was associated with hypersensitization to LPS.

**Table 3:** Histological evaluation according to the semi-quantitative system.

	Alteration in sinusoids	Vacuolization of hepatocytes	Erythrocyte congestion	Hepatocellular necrosis
LPS 1 h	1±0	1±0	1±0	1±0
G-CSF+LPS 1 h	2±1	2±0	2±1	1±1
G-CSF+LPS+LBPK95A 1 h	2±1	1±0	1±1	1±0
LPS 6 h	2±0	3±1	1±0	1±0
G-CSF+LPS 6 h	4±1	4±1	4±1	3±1
G-CSF+LPS+LBPK95A 6 h	3±1	1±1	2±1	2±1

### G-CSF Induced LBP Expression Enhanced Hepatic Inflammation through Increasing Hepatic Uptake of LPS and Upregulating Expression of LPS Receptors

**Figure 3:**
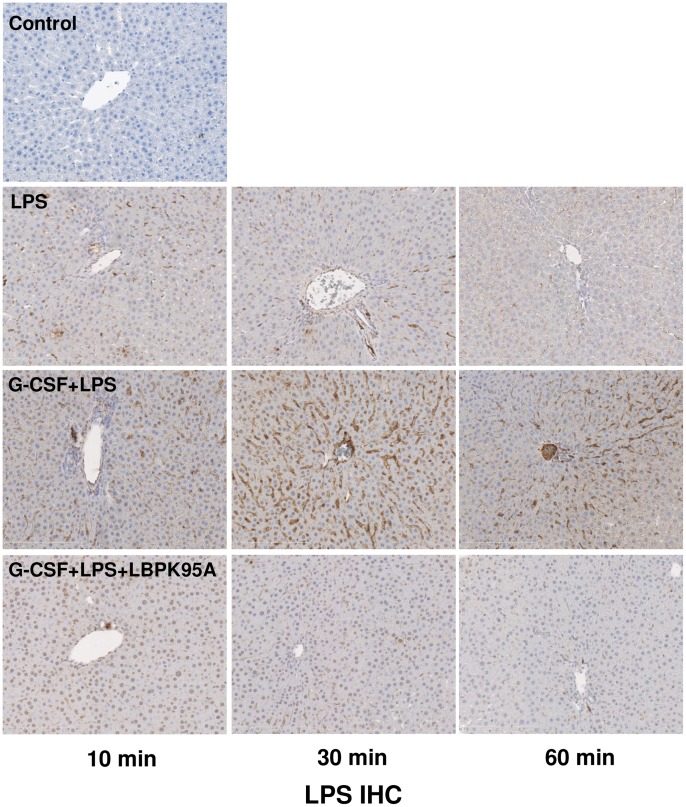
G-CSF induced LBP expression enhanced LPS-binding to the liver. LPS ICH staining was performed to assess the hepatic uptake of LPS. Enhanced LPS positive staining was observed in livers in G-CSF+LPS group. The increased hepatic uptake of LPS was inhibited by blockade of LBP. Original magnification ×200. Representative images from 4 rats per group were selected.

**Figure 4:**
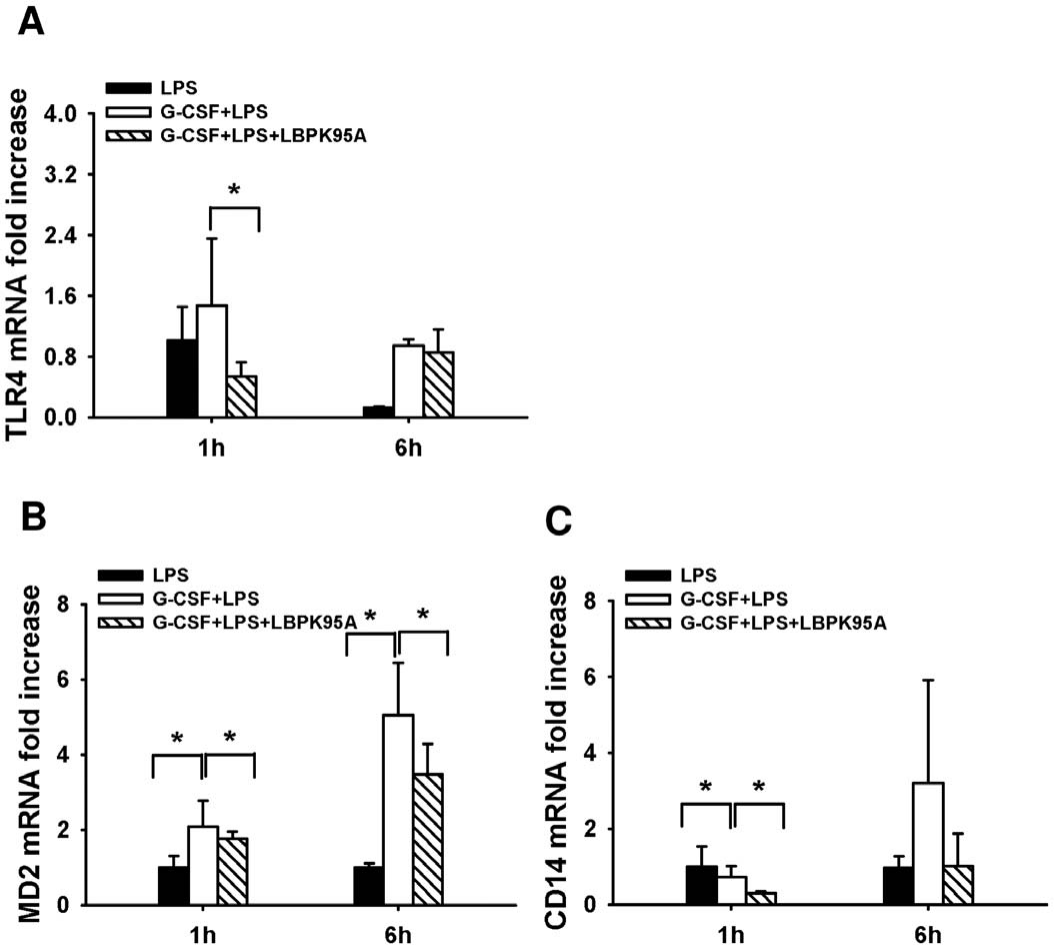
G-CSF induced LBP expression increased expression of other LPS receptors. (**A–C**) Hepatic mRNA expression of LPS receptors TLR-4, CD-14 and MD-2 was measured by quantitative PCR, respectively. G-CSF pretreatment caused significantly upregulation of TLR-4, CD-14 and MD-2 mRNA in G-CSF+LPS group. In contrast, blocking of LBP inhibited the upregulation in G-CSF+LPS+LBPK95A group. *p<0.05 Data are shown as mean ± SD, n = 6 per group.

**Figure 5:**
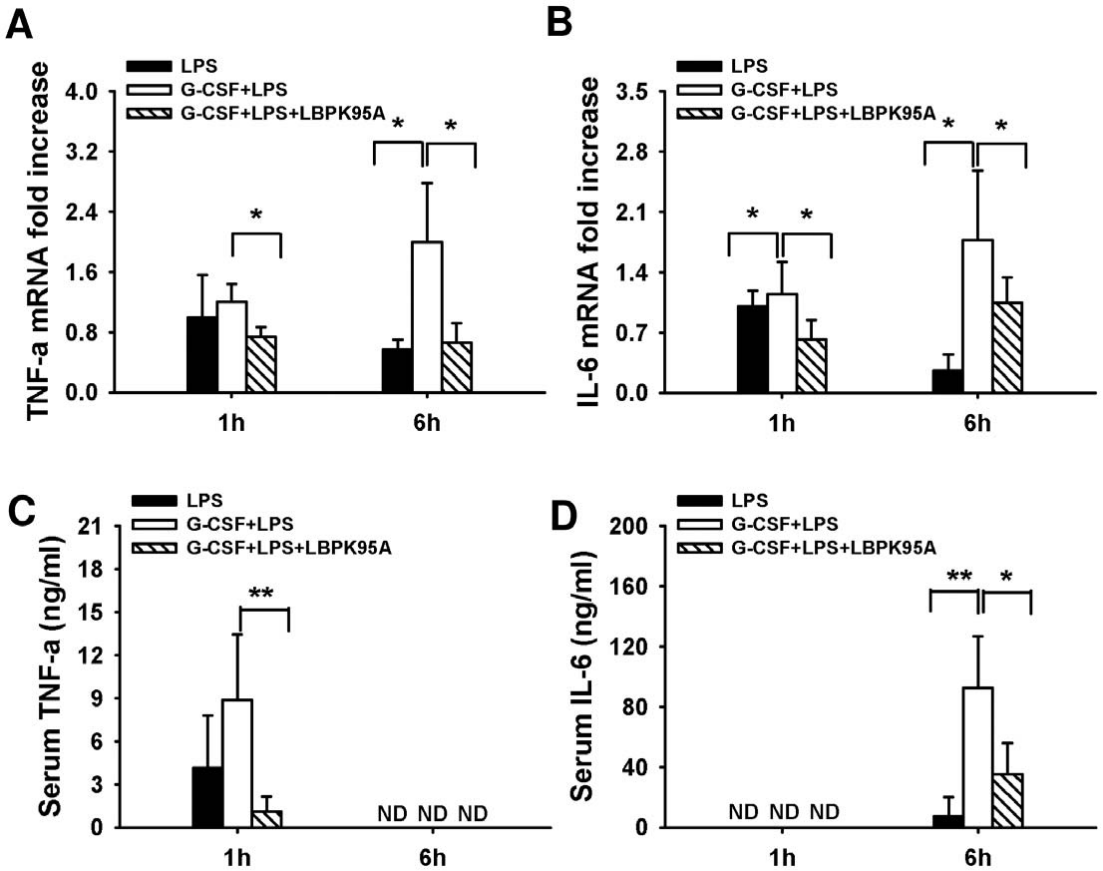
G-CSF induced LBP expression amplified the LPS induced inflammatory response. Hepatic and serum TNF-α, IL-6 levels were measured by quantitative PCR (A, B) and ELISA, respectively (C, D). Upregulation of LBP-levels via G-CSF prior to the LPS-challenge resulted in increased hepatic expression of TNF-α and IL-6 mRNA 1 h and 6 h after LPS injection. Blockade of LBP exhibited minimal increase in mRNA expression of these pro-inflammatory cytokines. Serum TNF-α and IL-6 levels were decreased at 1 h and 6 h, and had the similar pattern with the hepatic mRNA levels. *p<0.05, **p<0.01 Data are shown as mean ± SD, n = 6 per group.

To explore the mechanism through which elevation of LBP caused LPS sensitization, we investigated hepatic uptake of LPS expression of LPS receptors. It is reported that LBP is responsible for LPS recognition and transfer of LPS to TLR-4 complex [25]. We hypothesized that the G-CSF induced LBP expression might accelerate the LPS transfer to the liver, which might associate with the expression of LPS receptors and the initiation of the inflammatory response. By performing LPS IHC staining, we found that the hepatic uptake of LPS was visible within 10 min after LPS injection (Figure 3). The intensity of LPS staining in G-CSF+LPS group was significantly higher than in LPS group throughout the observation period. Interestingly, the hepatic uptake of LPS was dramatically inhibited by LBP blockade (Figure 3). To determine whether the enhanced LPS binding in the liver was associated with the activation LPS receptors, we compared the mRNA expression of hepatic TLR-4, MD-2 and CD-14. We demonstrated that the mRNA levels of TLR-4, MD-2 and CD-14 were significantly increased in G-CSF+LPS group 1 h after LPS administration (Figure 4A–C). Furthermore, the expression of these LPS receptors was inhibited by LBP blockade, indicated by significant decrease of hepatic TLR-4, CD-14 and MD-2 mRNA levels. The increased expression of LPS receptors resulted in augmenting of the inflammatory response, indicated by release of inflammatory cytokines, such as TNF-α and IL-6. Consistently, the hepatic mRNA expression of these cytokines was significantly increased at 1 h and 6 h after LPS administration in G-CSF+LPS groups. Furthermore, animals that were treated with LBP-inhibitory peptide exhibited lower increase in hepatic TNF-α and IL-6 hepatic mRNA expression levels (Figure 5A, B). Strong correlation between the hepatic mRNA expression levels and serum levels was observed, although the serum TNF-α and IL -6 was only detectable at 1 h and 6 h, respectively (Figure 5C, D), Taken together, these findings suggested that the G-CSF induced LBP expression mediated LPS transfer to the liver and associated with the initiation of inflammatory response.

### G-CSF Induced LBP Expression Associates with Hepatic Neutrophil Infiltration and Monocyte Recruitment into the Liver

**Figure 6:**
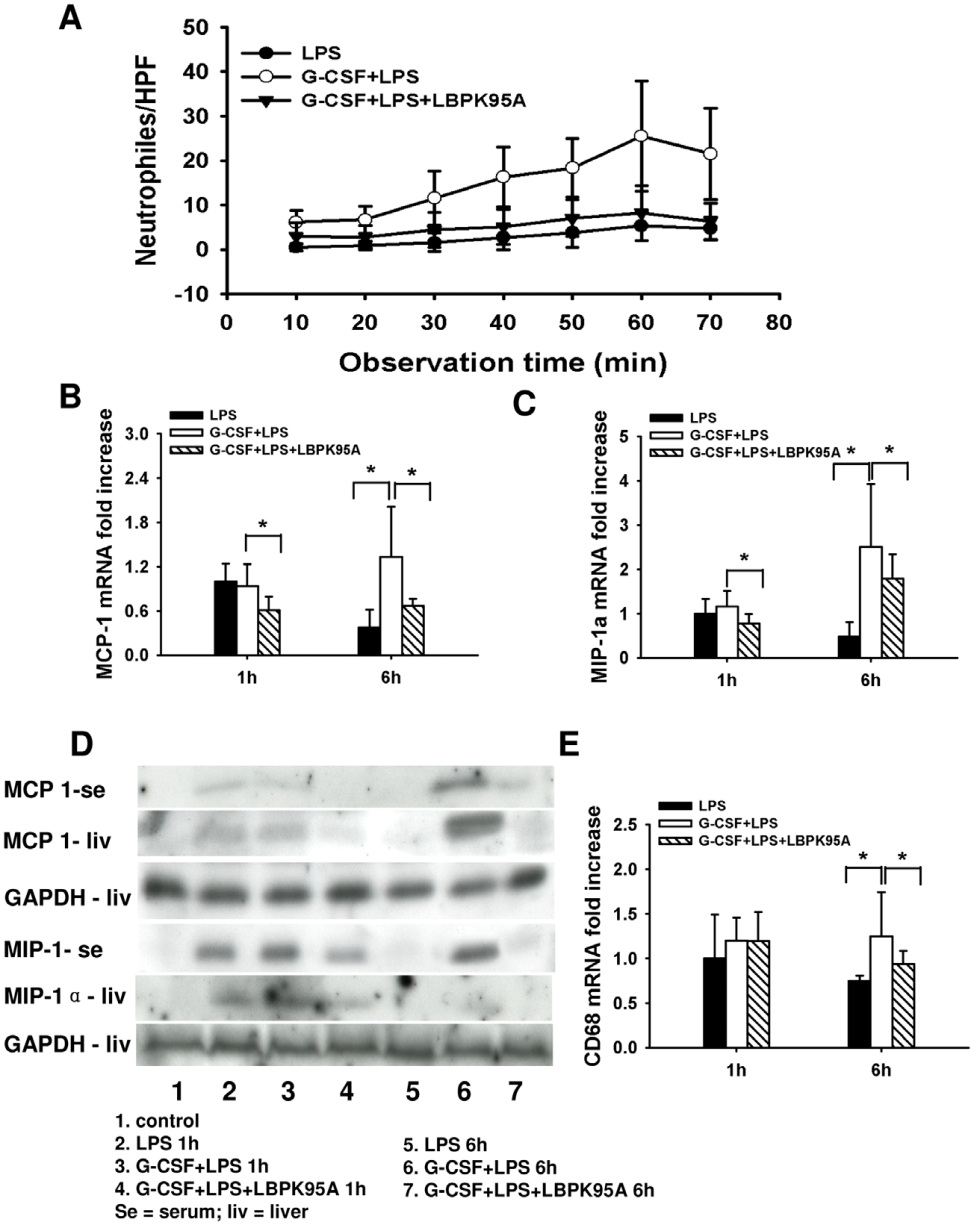
G-CSF induced LBP expression mediated neutrophil infiltration and monocytes recruitment. (**A**) The influx of neutrophil was analyzed by counting ASDCL positive cells. The neutrophil infiltration to liver was promoted by G-CSF pretreatment, but inhibited by blockade of LBP by LBPK95A. (**B–D**) The expression of MCP-1 and MIP-1α mRNA and protein levels in liver tissues and serum. G-CSF pretreatment caused upregulation of MCP-1 and MIP-1α expression and release in G-CSF+LPS group. The upregulation was attenuated by blockade of LBP in G-CSF+LPS+LBPK95A group. (**E**) The monocytes recruitment levels were indicated by hepatic CD68 mRNA expression. G-CSF pretreatment increased hepatic CD68 expression at 6 h, which was decreased after LBP blockade. *p<0.05 Data are shown as mean ± SD, n = 6 per group.

The influx of neutrophils to the inflammatory site is considered to be an essential part of the host defence to infection. This drew our attention to explore three potential reasons: (1) G-CSF pretreatment induced neutrophil proliferation; (2) G-CSF pretreatment upregulated LBP expression in BMC, which was the main source of mature neutrophils (3) LBP might mediate early neutrophil infiltration in inflammatory response [26]. Therefore, we hypothesized that the neutrophil infiltration was LBP mediated. We analyzed the ASDCL positive stained cells for this purpose. In G-CSF+LPS group, an increased neutrophil infiltration was observed as early as 20 min after LPS injection (Figure 6). The maximal effect was observed 50 min after LPS injection. At that point, the influx neutrophil number increased to about 6 fold over LPS group. The increase of neutrophil infiltration was LBP mediated. After blockade of LBP, the neutrophil infiltration was inhibited, indicated by a decrease in the influx cell number in G-CSF+LPS+LBPK95A group.

After arriving at sites of infection, neutrophils secrete cytokines and chemokines such as MCP-1 and macrophage inflammatory protein MIP-1α for monocytes recruitment [27]. In addition to the effect on neutrophil infiltration, we here also clarified whether monocytes recruitment was affected. We found that the hepatic mRNA and proteion expression levels of MCP-1 and MIP-1α was significantly increased in G-CSF+LPS group. In contrast, LBP blockade attenuated this effect of G-CSF pretreatment on monocytes recruitment (Figure 6B–D). To confirm this, we detected the MCP-1 and MIP-1α levels in serum. As shown in Figure 6D, serum MCP-1 and MIP-1α levels were highly associated with the hepatic mRNA and protein expression. The expression of chemotactic protein led to monocyte recruitment. The monocyte recruitment was indicated by hepatic CD68 mRNA expression. G-CSF pretreatment increased hepatic CD68 mRNA expression at 6 h. However, CD68 mRNA levels were decreased after LBP blockade. Take together, these data suggested that G-CSF induced LBP expression contributed to early neutrophil infiltration, as well as monocyte recruitment.

## Discussion

To the best of our knowledge, two independent findings were related for the first time in the present study. First observation: G-CSF induces LBP-upregulation and second observation: LBP upregulation sensitizes to a subsequent LPS-challenge. We demonstrated that G-CSF pretreatment sensitized to LPS, and this effect was mediated via upregulation of LBP. G-CSF pretreatment prior to the LPS-challenge caused an aggravated inflammatory response, severe hepatic damage and the death of the animals. Of note, the expression of inflammatory cytokines, liver damage and mortality was attenuated by blocking LBP using LBP inhibitory peptide, demonstrating the crucial role of LBP-upregulation in the pathogenesis of SIRS. Furthermore, we provided evidence that LPS-sensitization and aggravation of the inflammatory response seemed to be related to the enhanced binding of LPS in liver. Our data suggested that G-CSF induced LBP expression could be used as a new model of LBP related sensitization to LPS.

There is growing evidence that LBP expression sensitizes to LPS. Our data confirmed that LBP-levels determined the severity of inflammatory response to LPS. LBP blockade in the sensitization models reversed this effect (Table 4). Inflammatory response to LPS was reduced using an LBP inhibitory peptide [14] or an anti-LBP antibody respectively [15]. The same phenomenon was observed in two different models of LPS-sensitization: lack of LBP protected animals from galactosamine induced LPS-sensitization [14]. Similarly, animals were protected from galactosamine induced LPS-sensitization when applying an anti-LPS antibody prior to the LPS challenge [28]. Sensitization to LPS was also observed in wild-type mice after BDL and abolished in LBP-KO mice [4]. LBP blockade decreased hepatic injury and death following acetaminophen-induced liver injury [29]. Similar results were observed in hemorrhagic shock models, where the lack of LBP attenuated the hepatocelluar injury and inflammatory response in LBP-KO mice [30].

**Table 4:** Modulation of LPS-response via LBP blockade.

Author	LBP- upregulation	LBP blockade	Down-regulation of LBPwas associated with
Arena et al [14]	LPS	LBPK95A	Reduced inflammatory response
Le Roy et al [15]	LPS	LBP-antibody	Decreased TNF-α levels
Jack et al [16]	Galactosamine+LPS	LBP-KO	High survival rate
Gallay et al [28]	Galactosamine+LPS	LBP-antibody	Protective effect of mice from septic shock
Minter et al [4]	BDL	LBP-KO	High survival rate in BDL model
Lehnert et al [30]	Hemorrhagic-shock	LBP-KO	Low hepatocellular damage after hemorrhagic shock
Su et al [29]	Acetaminophen	LBP-KO	Low toxic effect of Acetaminophen

These findings complement the perception that LBP acts as a soluble ‘pattern-recognition molecule’. According to the pattern recognition theory, LBP transfers LPS to CD 14, and then triggers the inflammatory cascade though TLR-4 signalling pathway. Activation of the TLR-4 signal pathway initiates a cascade of events, including translocation of nuclear factor kappa B to the nucleus and leading to production and release of inflammatory cytokines [31]. In our experiment, G-CSF induced LBP expression was crucial for the enhanced LPS induced inflammatory response, indicated by the reversal of the effect when applying the inhibitory peptide.

We hypothesized that the sensitization to LPS may be mediated via enhanced LPS-binding in the liver. LPS uptake occurs mainly in the liver. Liver cells, such as Kupffer cells, hepatocytes and sinusoidal endothelial cells can uptake circulating LPS, both after ex-vivo exposure to LPS and in-vivo following LPS injection [32]–[34]. Our results confirmed that the liver is involved in LPS-uptake. We demonstrated, that upregulation of LBP was observed in liver after G-CSF pretreatment. Compared to control animals, upregulation of LBP in the liver was associated with an enhanced and rapid hepatic uptake of LPS and a massively enhanced inflammatory cytokines expression. These findings further confirmed that LBP acted as major mediator for recognition of LPS and initiation of the inflammatory response in the liver.

LBP expression was induced by G-CSF pretreatment. G-CSF is a hematopoietic cytokine that acts on neutrophil proliferation and differentiation. It is widely accepted to use G-SCF in neutropenic patients at high risk for infection and sepsis to enhance the host immune defence [35]. G-CSF was also evaluated in clinical trials for treatment and prophylaxis of sepsis, but no clear benefit was shown [21]. We hypothesized that the G-CSF pretreatment might not only facilitate neutrophil differentiation, but also augment the expression and eventually the release of LBP from neutrophils and liver tissue. We previously observed that G-CSF pretreatment increased hepatic LBP expression [18]. Here we confirmed and extended these observations. We found that G-CSF pretreatment upregulated both LBP-expression in bone morrow cells and increased LBP levels in serum.

Induction of LBP can have two effects. Our data suggested that the G-CSF induced LBP-expression could cause sensitization to LPS, indicated by increased hepatic uptake of LPS and an enhanced inflammatory response. However, our literature analysis revealed that LBP was also involved in the efficient elimination of bacteria, e.g. Kang K et al found that LBP deficient mice showed delayed neutrophils influx in case of a peritoneal infection [26]. This led to the conclusion that LBP might have a dual role: augmenting the inflammatory response to bacterial toxin such as LPS, and contributing to bacterial elimination via associate neutrophil infiltration. These two functions might help to explain that determination of LBP levels alone was not useful as biomarker of the severity of sepsis. It might also help to explain that G-CSF treatment in septic patients with an unknown level of circulating LPS may lead to controversial results.

In summary, G-CSF induced LBP expression could serve as a new model for investigation of LPS sensitization. G-CSF-induced LBP sensitization is mediated via an enhanced binding of LPS in the liver. Increased sensitivity to LPS after G-CSF treatment might help to explain the controversial results observed after G-CSF treatment in septic patients.

## Supporting Information

Figure S1 **Grading of semi-quantitative scoring system for histological evaluation.** Different Severities of sinusoid dilation and erythrocyte congestion in the semi-quantitative scoring system were indicated using pictures from H&E staining.
(TIF)

Table S1 **Primers used for quantitative PCR studies.**
(DOC)

## References

[1] Prins JM, van Deventer SJ, Kuijper EJ, Speelman P (1994) Clinical relevance of antibiotic-induced endotoxin release. Antimicrob Agents Chemother 38: 1211–1218. 8092816 10.1128/aac.38.6.1211 PMC188188

[2] Alexander C, Rietschel ET (2001) Bacterial lipopolysaccharides and innate immunity. J Endotoxin Res 7: 167–202. 11581570

[3] Mileski WJ, Winn RK, Harlan JM, Rice CL (1992) Sensitivity to endotoxin in rabbits is increased after hemorrhagic shock. J Appl Physiol 73: 1146–1149. 1400029 10.1152/jappl.1992.73.3.1146

[4] Minter RM, Bi X, Ben Josef G, Wang T, Hu B, et al (2009) LPS-binding protein mediates LPS-induced liver injury and mortality in the setting of biliary obstruction. Am J Physiol Gastrointest Liver Physiol 296: G45–G54. 18948440 10.1152/ajpgi.00041.2008 PMC2636928

[5] Galanos C, Freudenberg MA, Reutter W (1979) Galactosamine-induced sensitization to the lethal effects of endotoxin. Proc Natl Acad Sci U S A 76: 5939–5943. 293694 10.1073/pnas.76.11.5939 PMC411768

[6] Ferluga J, Allison AC (1978) Role of mononuclear infiltrating cells in pathogenesis of hepatitis. Lancet 2: 610–611. 80531 10.1016/s0140-6736(78)92828-3

[7] SUTER E (1962) Hyperreactivity to endotoxin in mice infected with BCG. Studies on the role of concomitant infection. J Immunol 89: 377–381. 13918520

[8] Cornelie S, Wiel E, Lund N, Lebuffe G, Vendeville C, et al (2002) Cytosine-phosphate-guanine (CpG) motifs are sensitizing agents for lipopolysaccharide in toxic shock model. Intensive Care Med 28: 1340–1347. 12209287 10.1007/s00134-002-1418-z

[9] Schumann RR, Leong SR, Flaggs GW, Gray PW, Wright SD, et al (1990) Structure and function of lipopolysaccharide binding protein. Science 249: 1429–1431. 2402637 10.1126/science.2402637

[10] Schumann RR, Zweigner J (1999) A novel acute-phase marker: lipopolysaccharide binding protein (LBP). Clin Chem Lab Med 37: 271–274. 10353471 10.1515/CCLM.1999.047

[11] Schroder NW, Opitz B, Lamping N, Michelsen KS, Zahringer U, et al (2000) Involvement of lipopolysaccharide binding protein, CD14, and Toll-like receptors in the initiation of innate immune responses by Treponema glycolipids. J Immunol 165: 2683–2693. 10946299 10.4049/jimmunol.165.5.2683

[12] Vollmer T, Piper C, Kleesiek K, Dreier J (2009) Lipopolysaccharide-binding protein: a new biomarker for infectious endocarditis? Clin Chem 55: 295–304. 18832474 10.1373/clinchem.2008.106195

[13] Sakr Y, Burgett U, Nacul FE, Reinhart K, Brunkhorst F (2008) Lipopolysaccharide binding protein in a surgical intensive care unit: a marker of sepsis? Crit Care Med 36: 2014–2022. 18552695 10.1097/CCM.0b013e31817b86e3

[14] Arana MJ, Vallespi MG, Chinea G, Vallespi GV, Rodriguez-Alonso I, et al (2003) Inhibition of LPS-responses by synthetic peptides derived from LBP associates with the ability of the peptides to block LBP-LPS interaction. J Endotoxin Res 9: 281–291. 14577844 10.1179/096805103225002520

[15] Le Roy D, Di Padova F, Tees R, Lengacher S, Landmann R, et al (1999) Monoclonal antibodies to murine lipopolysaccharide (LPS)-binding protein (LBP) protect mice from lethal endotoxemia by blocking either the binding of LPS to LBP or the presentation of LPS/LBP complexes to CD14. J Immunol 162: 7454–7460. 10358200

[16] Jack RS, Fan X, Bernheiden M, Rune G, Ehlers M, et al (1997) Lipopolysaccharide-binding protein is required to combat a murine gram-negative bacterial infection. Nature 389: 742–745. 9338787 10.1038/39622

[17] Wurfel MM, Monks BG, Ingalls RR, Dedrick RL, Delude R, et al (1997) Targeted deletion of the lipopolysaccharide (LPS)-binding protein gene leads to profound suppression of LPS responses ex vivo, whereas in vivo responses remain intact. J Exp Med 186: 2051–2056. 9396775 10.1084/jem.186.12.2051 PMC2199164

[18] Ji Y, Dahmen U, Madrahimov N, Madrahimova F, Xing W, et al (2009) G-CSF administration in a small-for-size liver model. J Invest Surg 22: 167–177. 19466653 10.1080/08941930802713027

[19] Pollmacher T, Korth C, Mullington J, Schreiber W, Sauer J, et al (1996) Effects of granulocyte colony-stimulating factor on plasma cytokine and cytokine receptor levels and on the in vivo host response to endotoxin in healthy men. Blood 87: 900–905. 8562960

[20] Lundblad R, Nesland JM, Giercksky KE (1996) Granulocyte colony-stimulating factor improves survival rate and reduces concentrations of bacteria, endotoxin, tumor necrosis factor, and endothelin-1 in fulminant intra-abdominal sepsis in rats. Crit Care Med 24: 820–826. 8706460 10.1097/00003246-199605000-00016

[21] Bo L, Wang F, Zhu J, Li J, Deng X (2011) Granulocyte-colony stimulating factor (G-CSF) and granulocyte-macrophage colony stimulating factor (GM-CSF) for sepsis: a meta-analysis. Crit Care 15: R58. 21310070 10.1186/cc10031 PMC3221991

[22] Liu A, Fang H, Dirsch O, Jin H, Dahmen U (2012) Oxidation of HMGB1 Causes Attenuation of Its Pro-Inflammatory Activity and Occurs during Liver Ischemia and Reperfusion. PLoS One 7: e35379. 22514737 10.1371/journal.pone.0035379 PMC3325960

[23] Leder LD (1970) Diagnostic experiences with the naphthol AS-D chloroacetate esterase reaction. Blut 21: 1–8. 4988077 10.1007/BF01633225

[24] Yano R, Matsuyama T, Kaneko T, Kurio H, Murayama E, et al (2010) Bactericidal/Permeability-increasing protein is associated with the acrosome region of rodent epididymal spermatozoa. J Androl 31: 201–214. 19745219 10.2164/jandrol.109.007880

[25] Schumann RR (2011) Old and new findings on lipopolysaccharide-binding protein: a soluble pattern-recognition molecule. Biochem Soc Trans 39: 989–993. 21787335 10.1042/BST0390989

[26] Yang KK, Dorner BG, Merkel U, Ryffel B, Schutt C, et al (2002) Neutrophil influx in response to a peritoneal infection with Salmonella is delayed in lipopolysaccharide-binding protein or CD14-deficient mice. J Immunol 169: 4475–4480. 12370383 10.4049/jimmunol.169.8.4475

[27] Amulic B, Cazalet C, Hayes GL, Metzler KD, Zychlinsky A (2012) Neutrophil function: from mechanisms to disease. Annu Rev Immunol 30: 459–489. 22224774 10.1146/annurev-immunol-020711-074942

[28] Gallay P, Heumann D, Le Roy D, Barras C, Glauser MP (1994) Mode of action of anti-lipopolysaccharide-binding protein antibodies for prevention of endotoxemic shock in mice. Proc Natl Acad Sci U S A 91: 7922–7926. 7520172 10.1073/pnas.91.17.7922 PMC44516

[29] Su GL, Gong KQ, Fan MH, Kelley WM, Hsieh J, et al (2005) Lipopolysaccharide-binding protein modulates acetaminophen-induced liver injury in mice. Hepatology 41: 187–195. 15619225 10.1002/hep.20533

[30] Lehnert M, Uehara T, Bradford BU, Lind H, Zhong Z, et al (2006) Lipopolysaccharide-binding protein modulates hepatic damage and the inflammatory response after hemorrhagic shock and resuscitation. Am J Physiol Gastrointest Liver Physiol 291: G456–G463. 16614372 10.1152/ajpgi.00480.2005

[31] Cotroneo TM, Nemzek-Hamlin JA, Bayliss JM, Su GL. (2012) Lipopolysaccharide binding protein inhibitory peptide alters hepatic inflammatory response post-hemorrhagic shock. Innate Immun. 10.1177/1753425912444641 22535680

[32] Yasui M, Nakao A, Yuuki T, Harada A, Nonami T, et al (1995) Immunohistochemical detection of endotoxin in endotoxemic rats. Hepatogastroenterology 42: 683–690. 8751235

[33] van Oosten M, van Amersfoort ES, van Berkel TJ, Kuiper J (2001) Scavenger receptor-like receptors for the binding of lipopolysaccharide and lipoteichoic acid to liver endothelial and Kupffer cells. J Endotoxin Res 7: 381–384. 11753207 10.1177/09680519010070050601

[34] Scott MJ, Liu S, Shapiro RA, Vodovotz Y, Billiar TR (2009) Endotoxin uptake in mouse liver is blocked by endotoxin pretreatment through a suppressor of cytokine signaling-1-dependent mechanism. Hepatology 49: 1695–1708. 19296467 10.1002/hep.22839 PMC2677122

[35] Moore MA (1991) The clinical use of colony stimulating factors. Annu Rev Immunol 9: 159–191. 1910675 10.1146/annurev.iy.09.040191.001111

