# Supplementary figures and images for: Granulocyte Colony Stimulating Factor Induces Lipopolysaccharide (LPS) Sensitization via Upregulation of LPS Binding Protein in Rat
Source: PLoS One. 2013 Feb 20;8(2):e56654. doi: 10.1371/journal.pone.0056654 (PMC3577878; doi:10.1371/journal.pone.0056654)

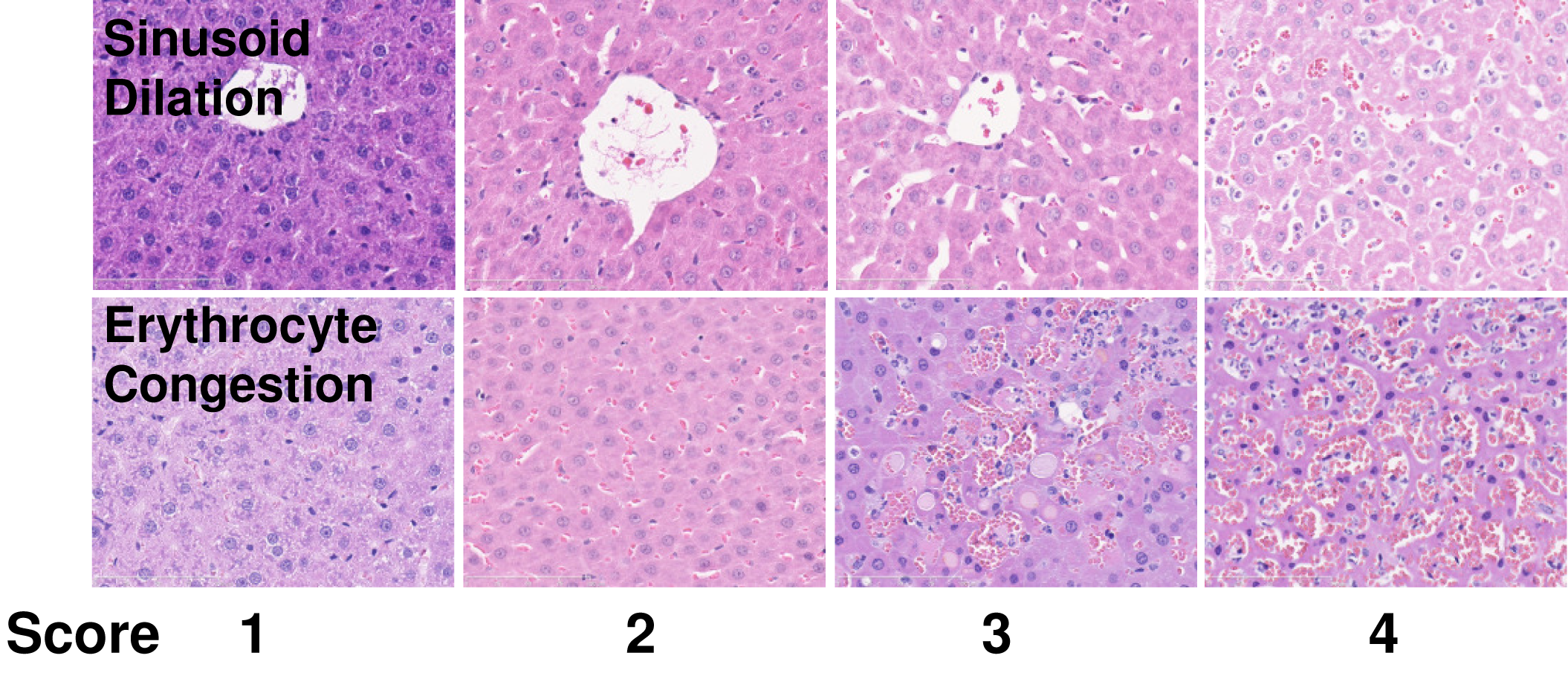

Supplement: Figure S1 — Grading of semi-quantitative scoring system for histological evaluation. Different Severities of sinusoid dilation and erythrocyte congestion in the semi-quantitative scoring system were indicated using pictures from H&E staining. (TIF) [file pone.0056654.s001.tif]
