# Supplementary material for: Granulocyte Colony Stimulating Factor Induces Lipopolysaccharide (LPS) Sensitization via Upregulation of LPS Binding Protein in Rat
Source: PLoS One. 2013 Feb 20;8(2):e56654. doi: 10.1371/journal.pone.0056654 (PMC3577878; doi:10.1371/journal.pone.0056654)
Supplement: Table S1 — Primers used for quantitative PCR studies. (DOC) [file pone.0056654.s002.doc]

**Table S1 Primers used for quantitative PCR studies**

| **Gene** |  | **Sequence (5’ - 3’)** | **Probe**^a^ |  | **GeneBank**  **(Rattus norvegicus)** |
| --- | --- | --- | --- | --- | --- |
| LBP | F | ATCCGGCTGAACACCAAG | #82 | 69 | NM_017208.2 |
|  | R | TGTCGGGGTACTTTCTGGTT |  |  |  |
| BPI | F | tccgcatcaaagtctctgg | #83 | 62 | NM_001004079.1 |
|  | R | aattttcctgtggaacagttgg |  |  |  |
| TNF-α | F | TGAACTTCGGGGTGATCG | #63 | 122 | NM_012675 |
|  | R | GGGCTTGTCACTCGAGTTTT |  |  |  |
| IL-6 | F | CCTGGAGTTTGTGAAGAACAACT | #106 | 142 | NM_012589 |
|  | R | GGAAGTTGGGGTAGGAAGGA |  |  |  |
| IL-10 | F | AGTGGAGCAGGTGAAGAATGA | #125 | 62 | NM_012854.1 |
|  | R | TCATGGCCTTGTAGACACCTT |  |  |  |
| IL-1ß | F | GCTGACAGACCCCAAAAGAT | #117 | 74 | NM_031512.2 |
|  | R | AGCTGGATGCTCTCATCTGG |  |  |  |
| TLR4 | F | ggatgatgcctctcttgcat | #95 | 127 | NM_019178.1 |
|  | R | tgatccatgcattggtaggtaa |  |  |  |
| CD14 | F | aaagaaactgaagcctttctcg | #26 | 90 | NM_021744.1 |
|  | R | agcaacaagccgagcataa |  |  |  |
| MD2 | F | tgatgattattctttttgcagagc | #75 | 127 | AY963291.1 |
|  | R | atccccagcaatggcttc |  |  |  |
| MCP-1 | F | agcatccacgtgctgtctc | #62 | 72 | NM_031530.1 |
|  | R | gatcatcttgccagtgaatgag |  |  |  |
| MIP-1a | F | gcgctctggaacgaagtct | #40 | 82 | NM_013025.2 |
|  | R | gaatttgccgtccataggag |  |  |  |
| CD68 | F | acggacagcttacctttgga | #21 | 118 | NM_001031638.1 |
|  | R | aatgtccactgtgctgcttg |  |  |  |
| HPRT | F | GACCGGTTCTGTCATGTCG | #95 | 61 | NM_012583 |
|  | R | ACCTGGTTCATCATCACTAATCAC |  |  |  |

^a^Universal ProbeLibrary probes
